# Frequency-specific intermuscular coherence of synergistic muscles during an isometric force generation task

**DOI:** 10.3389/fncir.2025.1675012

**Published:** 2025-11-07

**Authors:** Daniele Borzelli, Alberto Cacciola, Carlo Vittorio Cannistraci, Angelo Alito, Demetrio Milardi, Andrea d’Avella

**Affiliations:** 1 Brain Mapping Lab, Department of Biomedical, Dental Sciences and Morphological and Functional Imaging, University of Messina, Messina, Italy; 2 Laboratory of Neuromotor Physiology, IRCCS Fondazione Santa Lucia, Rome, Italy; 3 Department of Biomedical Sciences, Humanitas University, Pieve Emanuele/Milan, Italy; 4 IRCCS Humanitas Research Hospital, Rozzano/Milan, Italy; 5 Tsinghua Laboratory of Brain and Intelligence (THBI), Department of Psychological and Cognitive Sciences, Center for Complex Network Intelligence (CCNI), Tsinghua University, Beijing, China; 6 Department of Computer Science and Technology, Tsinghua University, Beijing, China; 7 School of Biomedical Engineering, Tsinghua University, Beijing, China; 8 Department of Biology, University of Rome Tor Vergata, Rome, Italy

**Keywords:** motor modules, tri-dimensional force, alpha band, low-beta band, muscle-muscle coherence, muscle coordination, frequency layers, non-negative matrix factorization

## Abstract

**Introduction:**

Motor tasks require the flexible selection and coordination of multiple muscles, which may be achieved through the organization and combination of muscle synergies. Although multiple muscles may receive a shared neural drive, and each muscle may also receive distinct neural inputs, there is ongoing debate about whether synergies accurately reflect shared neural drives. This study aimed to compare the spectral characteristics of the common drive shared among muscles within the same synergy to those shared among muscles belonging to different synergies.

**Methods:**

Electromyographic signals were recorded from upper limb muscles during an isometric multi-directional force generation task. Synergies were identified using non-negative matrix factorization (NMF), and coherence analysis was conducted to evaluate common drives among muscles within and across synergies. A methodological limitation of previous studies was to segment muscle activity into standard frequency bands. Here, we overcome it by proposing to automatically detect subject-specific and physiologically relevant frequency layers. The application of NMF on the coherence spectra of muscle pairs as a method for automatically detecting physiologically relevant frequency bands sheds light into the neural basis of muscle coordination.

**Results:**

Six frequency layers were identified, and muscle recruited within the same synergy showed a higher coherence within layers in the delta, alpha, and low-beta bands.

**Discussion:**

Our findings enhance the understanding of physiological mechanisms of motor coordination by elucidating the relationship between muscle synergies and the spectral characteristics of intermuscular coherence.

## 1 Introduction

A long-standing hypothesis suggests that muscle activations are coordinated through the organization of muscle synergies. The existence of muscle synergies has been supported by the observation of low dimensionality in the muscle patterns during several tasks including reaching and grasping (D’Avella et al., 2006; Overduin et al., 2008), postural control (Ting and Macpherson, 2005; Torres-Oviedo and Ting, 2007), locomotion (Ivanenko et al., 2004; Dominici et al., 2011; Rimini et al., 2017), and isomeric force generation (Borzelli et al., 2013; Gentner et al., 2013), and muscle synergies may be affected by neurological lesions (Zhao et al., 2023; Borzelli et al., 2024a). Yet, different aspects of synergistic organization may emerge at different neural levels, including spinal (Takei and Seki, 2010), cortical (Overduin et al., 2012), and cerebellar (Berger et al., 2020) circuits. While the neural origin of muscle synergies has been supported through different approaches (Tresch and Jarc, 2009; Overduin et al., 2012; Berger et al., 2013, 2022; Bizzi and Cheung, 2013; Cheung and Seki, 2021), the neural mechanisms underlying the coordination of multiple muscles at different levels are still unclear. While the synergy hypothesis focuses on how the central nervous system coordinates groups of muscles to cope with the redundancy of the musculoskeletal system, studies on common synaptic input investigate the neural implementation of this coordination through shared inputs to motoneuron pools. Several studies support the existence of common synaptic inputs that drive different muscles (De Luca and Mambrito, 1987; Kilner et al., 1999; Laine et al., 2015; Hug et al., 2022; Borzelli et al., 2024b). Although both frameworks posit a common command driving multiple muscles, they operate at different descriptive levels and lead to distinct empirical predictions: the synergy hypothesis predicts a functional invariant activation ratio among muscles, whereas the common-input framework predicts correlated neural activity, measurable as intermuscular coherence. Therefore, to link the two levels, it is reasonable to hypothesize that the coordinated activation of multiple muscles within synergies depends on the existence of common synaptic inputs. Indeed, recent studies demonstrated that the activity of the muscles recruited within the same synergy shows a significant synchronous modulation (Danna-Dos-Santos et al., 2014; De Marchis et al., 2015; Frère, 2017; Laine et al., 2021). However, these studies examined the activity of only a few muscles during a limited set of conditions (Danna-Dos-Santos et al., 2014) or investigated the occurrence of synchronization only in the 8 – 16 Hz band (Laine et al., 2021), thus limiting the understanding of the neural architecture underlying the synergistic recruitment of muscles. Although low-frequency components of the common inputs to motor neurons represent the effective drive that controls the exertion of force (Farina et al., 2014), a synaptic input from corticospinal neurons may occur at higher frequencies (Farmer et al., 1997). In fact, significant cortico-synergy coherence was recently identified in high-frequency bands (Zandvoort et al., 2019; Ortega-Auriol et al., 2023). Moreover, studies performed on a dynamic task (De Marchis et al., 2015) demonstrated high-frequency synchronous modulation but only between a few pairs of muscles recruited by the same synergies, and only when a functional force production was required. Previous work, therefore indicates that muscle coordination relies on frequency-specific neural drives, but the precise functional role and consistency of these frequency bands across individuals remain unclear.

An essential factor in determining the role of physiological frequency bands is the identification of their limits. Unfortunately, there is no consensus in the literature on the definition of band limits. The upper limit of the alpha band was set in different studies to 12 Hz (Mehrkanoon et al., 2014), 13 Hz (Yavuz et al., 2015; Leonardi et al., 2022), or 15 Hz (De Marchis et al., 2015). The boundary between low- to high-beta bands was set to 20 Hz (Shim et al., 2021), 21 Hz (van Wijk et al., 2017), or 25 Hz (Witte et al., 2007), while the beta upper limit was set to 25 Hz (Brinkman et al., 2014), 30 Hz (De Marchis et al., 2015; Farina and Negro, 2015) or 35 Hz (Del Vecchio et al., 2019). These inconsistencies make it difficult to interpret how specific frequency components contribute to muscle coordination and to compare results across studies.

To address this gap, we aimed to investigate whether muscles grouped within the same synergy exhibit stronger synchronization across physiologically relevant frequency bands than muscles belonging to different synergies. In particular, we adopted a data-driven, subject-specific approach to define frequency bands based on intermuscular coherence structure (Boonstra et al., 2015), which may help clarify how shared neural drives at different frequencies shape synergistic muscle activations. This data-driven approach may improve the alignment of spectral features across subjects and enable applications to patient populations, where frequency content may be shifted or distorted (Houston et al., 2021). This approach was tested during the generation of submaximal isometric force at the hand in multiple directions, while collecting the electromyographic activity from multiple muscles acting on the shoulder and elbow joints.

We hypothesized that if muscle synergies are implemented through common neural inputs, muscles within the same synergy must show higher intermuscular coherence than those recruited in different synergies, especially within frequency bands relevant for isometric force generation. Therefore, investigating changes in the intermuscular coherence at specific frequency bands would reveal functional neural mechanisms

This work therefore provides new insight into the neural organization of muscle synergies by disentangling the frequency-specific components of shared motor input while avoiding a priori assumptions about fixed frequency band limits.

## 2 Materials and methods

Participants, experimental setup, and experimental protocols are briefly described below. More details can be found in a previous paper (Borzelli et al., 2013) presenting a different analysis of the same data set.

### 2.1 Participants

Nine right-handed participants (four females, mean age 29.6 ± 4.4 years, age range 24–39) took part in the experiment after giving written informed consent. All procedures were approved by the Ethical Review Board of Fondazione Santa Lucia (Prot. CE/AG4-PROG.222-34). All participants had no known neuromuscular disorder or recent injury of the right arm.

One participant was excluded from the analysis after realizing the occurrence of muscle activity also when he was asked to relax his muscles (i.e., during the relaxation phase at the beginning of each trial, see section “2.3 Experimental protocol”), with the consequent reduction of the performance likely due to fatigue.

### 2.2 Experimental setup

**FIGURE 1:**
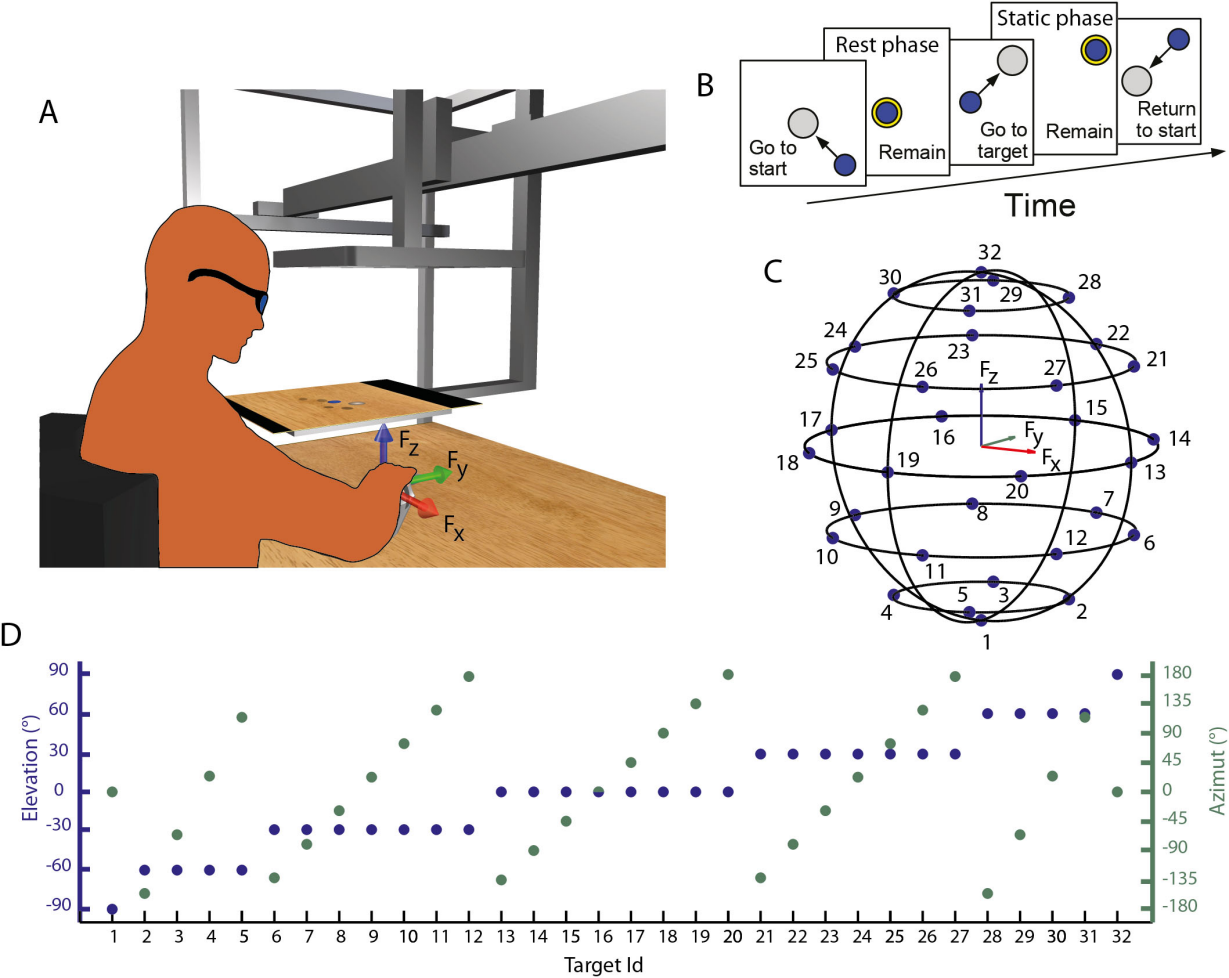
**(A)** Experimental setup. Participants sat on an isometric setup, with the right hand fixed in an orthosis rigidly connected to a force transducer. The red, green, and blue arrows represent the directions of the force axes, as collected from the transducer. Participants, wearing 3D glasses, look through a mirror at a 3D scene projected by a monitor, placed at the height of their eyes, and can control the displacement of a virtual cursor by applying force at the force transducer. **(B)** Task description. Participants were instructed to perform a center-out reaching task in which they had to maintain the cursor in a central start location for 3 s (rest phase), then reach a target and maintain the cursor within the target for 3 s (static phase). **(C,D)** Force targets. Positions of the 32 targets in the tri-dimensional force space **(C)** and their elevation and the azimuth angles **(D)**.

Participants sat on a racing car seat with their torso immobilized by safety belts and their right hand and forearm immobilized by a splint rigidly connected to a six-axis force transducer (Delta F/T Sensor, ATI Industrial Automation, Apex, NC, United States). In this posture (Figure 1A), the center of the palm was aligned with the body midline at the height of the sternum, and the elbow was flexed approximately by 90°. The force transducer was mounted under a desktop, whose height and distance from the participants could be adjusted according to the participants’ body size. Participants had the view of their right hand occluded by a mirror reflecting the image displayed by a 21-inch LCD monitor (Syncmaster 2233, Samsung Electronics Italia S.p.A., Cernusco sul Naviglio, MI, Italy), parallel to the desktop (Figure 1A). During the experiments, participants wore 3D shutter glasses (3DVision P854, NVIDIA Corporation, Santa Clara, CA, United States) to stereoscopically view a virtual desktop matching the real desktop and a spherical cursor whose displacement from an initial position was proportional to the three-dimensional force collected by the force transducer. The virtual scene was rendered by a 3D graphics card (QuadroFx3800, NVIDIA) and updated at 60 Hz. Cursor motion was simulated in real time as a mass accelerated by the force applied by the participant on the splint and adjusted adaptively in the range of 15–140 g as a sigmoidal function of the rate of change in the magnitude of the recorded force (Berger et al., 2013), a viscous force, and an elastic force proportional to the distance from the rest position.

Active surface bipolar electrodes (DE2.1, DelsysInc., Boston, MA) recorded the electromyographic (EMG) activity from 17 muscles acting on the right shoulder and elbow: teres major (TeresMaj), infraspinatus (InfraSp), latissimus dorsi (LatDorsi), inferior trapezius (TrapInf), middle trapezius (TrapMid), superior trapezius (TrapSup),brachioradialis (BracRad), biceps brachii long head (BicLong) and short head (BicShort), triceps brachii lateral head (TriLat), long head (TriLong), and medial head(TriMed), anterior deltoid (DeltA), middle deltoid (DeltM), posterior deltoid (DeltP), and pectoralis major clavicular (PectClav), and sternal (PectStern). Electrodes were placed in correspondence to the muscle belly (Hermens et al., 1999), and their correct placement was verified by observing the activation of each muscle during specific maneuvers. Force and EMG data were digitilized at 1kHz through an A/D PCI board (PCI-6229, National Instrument, Austin, TX, United States). Only force components (Fx lateral direction on the horizontal plane, positive to the right; Fy frontal direction on the horizontal plane, positive away from the chest; Fz vertical direction, positive up) were used during this experiment.

### 2.3 Experimental protocol

After an initial familiarization, each participant was instructed to exert a maximal force along 20 directions, two repetitions each, corresponding to the vertices of a dodecahedron centered in the origin. The mean value among the maximum force collected during each trial was identified as the Maximum Voluntary Force (MVF) value and used to scale the force required to reach the targets in the following block. The maximum activation of each muscle, identified during this block, was also retained as the Maximum Voluntary Contraction (MVC).

Then, participants were instructed to exert isometric forces to displace a virtual spherical cursor from an initial rest position to a target (Figure 1B). Targets, arranged on horizontal planes at different heights (Figures 1C, D), were approximately uniformly distributed on the surface of a sphere with a radius of 0.2 MVF and required the generation of forces along 32 directions, five repetitions each for a total of 160 trials. After an initial phase, in which participants were instructed to relax their muscles without exerting force (rest phase, 0.3 s), a target, indicated by a gray transparent sphere with a radius larger than the cursor sphere radius by 2% MVF, was displayed in one of the 32 locations. Therefore, participants were instructed to move and maintain the cursor within the target for 3s (hold phase) to successfully end the trial.

### 2.4 Data analysis

Electromyographic data were visually inspected to exclude those trials with signal artifacts. Trials in which the participant could not reach or remain within the target were also excluded from the analysis.

For each participant, NMF was first applied to EMG signals to identify muscle synergies. Then, NMF was also applied to the coherence between all pairs of muscles to determine the frequency bands at which muscles showed synchronous modulation (Boonstra et al., 2015). Coherence between muscles recruited within the same synergy and coherence between pairs of muscles that were never recruited within the same synergy were analyzed separately and compared within each subject-specific frequency band.

#### 2.4.1 Muscle synergies extraction

Raw EMG data were rectified, digitally low-pass filtered (2nd order Butterworth, 5 Hz cutoff), and re-sampled at 100 Hz to reduce data size. Mean EMG activity recorded during the rest phase of each trial was subtracted from data collected during the hold phase and EMG signals were normalized to the MVC. Muscle synergies of each participant were identified by the NMF algorithm (Lee and Seung, 1999), implemented in Matlab, from the pre-processed EMG signals averaged over time samples within the hold phase of each trial. Therefore, each muscle activation sample *m*_*m*_(*t*) was reconstructed as the combination of a unique set of spatial or time-invariant synergies *W_m_* scaled by time-varying synergy activation coefficients *c*_*m*_(*t*). Subscript *m* refers to the use of non-negative matrix factorization on *muscle* activation samples to extract muscle synergies, in contrast with subscript *c* that refers to the use of non-negative matrix factorization on coherence spectra between muscles to separate coherence between muscle pairs into frequency layers (see below).

The extraction algorithm was repeated 10 times for each number of synergies (1–17, i.e., the number of muscles) and the repetition with the highest fraction of total data variation explained by the synergy model, calculated as *R*^2^ = 1−SSE/SST, where SSE is the squared model residual and SST is the squared residual with respect to the mean EMG vector, was retained. The number of synergies *N* was chosen considering (i) the smallest *N* for which the *R*^2^ was larger than 0.9, (ii) as the point at which the *R*^2^ vs. *N* curve had a change in slope (detected as the first *N* at which MSE error of linear fit from *N* to 17, the number of muscles, was below 10−4), (iii) in case of mismatch between the number of synergies selected according to the two criteria, we chose the set of synergies with a more uniform distribution of preferred directions of the synergy activation coefficients (the direction of the maximum of the cosine function best fitting the directional tuning).

#### 2.4.2 Intermuscular coherence

The preprocessing for coherence calculation did not include any bandpass filtering of the EMG signal to avoid effects in the coherence analysis (Castronovo et al., 2018). The rectified raw EMG signal was initially demodulated by removing the slow-varying amplitude components to assure the stationarity requirement of coherence estimation (Carter, 1987). The demodulation was based on the Hilbert transform (Boonstra and Breakspear, 2012; Castronovo et al., 2018) for which the instantaneous frequency was calculated as in Equation 1:


$$\vartheta \left(t\right)=t\,a\,n^{-1}\,\left[\frac{x_{H}\,\left(t\right)}{x\,\left(t\right)}\right]$$ (1)

where *x* (*t*) was the EMG signal and *x*_*H*_ (*t*) its Hilbert transform, while the demodulated EMG signal was calculated as in Equation 2:


$${\text{x}}_{\text{D}}\left(\text{t}\right)=c\,o\,s\,\left[\vartheta \left(t\right)\right]$$ (2)

Then the mean activity was subtracted by the demodulated EMG signal of each muscle.

Since spurious contributions may occur in the absence of signals, for each trial, the coherence analysis was only calculated on pairs of muscles that were simultaneously active. The baseline noise was calculated as the variance of the EMG signal during the rest phase, when all muscles were expected to be relaxed. Then, a moving time window, lasting as the rest phase (i.e., 3 s), was used to determine the variance of the signal collected during the static phase. At each time step of the static phase, a muscle showing a variance at least 50 % higher than the variance calculated during the rest phase was identified as active during that time step. A muscle that was active in at least 75% of the time steps of the static phase was considered to be recruited during that trial.

The coherence was then calculated for all pairs of recruited muscles during the static phase as follows. The Welch’s power spectral density of each signal (*p*_*xx*_ and *p*_*yy*_) and the cross-power spectral density between the two signals (*p*_*xy*_), were calculated in a Hamming time window of 0.2 s with 50% overlap, as proposed in the literature (Boonstra et al., 2015). The Matlab functions “pwelch” and “cpsd” were implemented to calculate Welch’s power spectral density and the cross-power spectral density, respectively. The coherence was calculated as in Equation 3:


$$c_{x\,y}=\frac{p_{x\,y}\cdot p_{x\,y}^{*}}{p_{x\,x}\cdot p_{y\,y}}$$ (3)

where [⋅]* indicates the conjugate. The coherence contributions were then normalized by the Fischer transformation to allow for comparisons among different participants and it was calculated as in Equation 4:


$$Z_{x\,y}=\sqrt{2\,N_{s}}\,t\,a\,n\,h^{-1}\,\left(C_{x\,y}\right)$$ (4)

where *N*_*s*_ was the number of windowed segments used for the estimation of the coherence profile.

The significance of the estimated coherence spectra between each pair of muscles was assessed by employing a bootstrapping approach to the complex-valued cross-spectral density through phase randomization (Hurtado et al., 2004). Surrogates of the demodulated EMG signal of a muscle were generated by calculating the Fourier transform of the signal, independently shuffling the phase components, and then calculating the inverse Fourier transform back. This procedure ensures the preservation of the power spectrum of each signal but makes the two series completely uncorrelated in the frequency domain. For each trial, 100 surrogates were calculated for each active muscle, to calculate a set of coherence spectra expected from chance. Frequency bins showing lower coherence than the significance threshold, established at the 95% percentile of the bootstrap distribution, were set to zero. The coherence between pairs of muscles that were not simultaneously active was also set to zero.

The coherence spectra across the frequency range of 1–60 Hz (f frequency bins), over trials (t) and muscle pairs (m pairs), were concatenated to obtain a matrix with f rows and t × m columns, which was decomposed by NMF algorithm, assuming k modes with *k* = 1,⋯,f, where the number of frequency bins f in which the coherence spectra were separated depends on the window length and was 15 in this study. The decomposition defined two matrices: W_c_ and C_c_. The matrix W_c_ (f rows and k columns) represented the coherence patterns, and C_c_ (k rows and t × m columns) represented the edge weights. The k modes separated the frequencies into different layers. In this study, different W_c_ and C_c_ matrices were separately calculated from the data collected from each participant.

Previous studies identified the number of frequency layers by assuming a threshold in the data explained by the model. However, such thresholds were arbitrary (Kerkman et al., 2020; Houston et al., 2021), and the identified number of layers was influenced by the data pre-processing (Santuz et al., 2017). Therefore, in this study, we selected the number of layers by identifying the local minimum in the standard deviation of the coherence patterns *W_c_* across participants, which we used as a criterion to determine the optimal number of layers. This approach is physiologically motivated: a local minimum indicates a frequency separation that is most consistent across individuals, reflecting stable, participant-independent spectral features likely corresponding to functionally relevant neural drives. By using this criterion, we reduce the influence of inter-subject variability and avoid arbitrary thresholds, thus identifying layers that better capture the underlying physiological organization of muscle coordination.

#### 2.4.3 Coherence within synergistic and non-synergistic muscles

Our goal was to determine whether muscles recruited within the same synergy, which are extracted from the EMG activations, showed a higher synchronous modulation than muscles that were never recruited within the same synergy.

**FIGURE 2:**
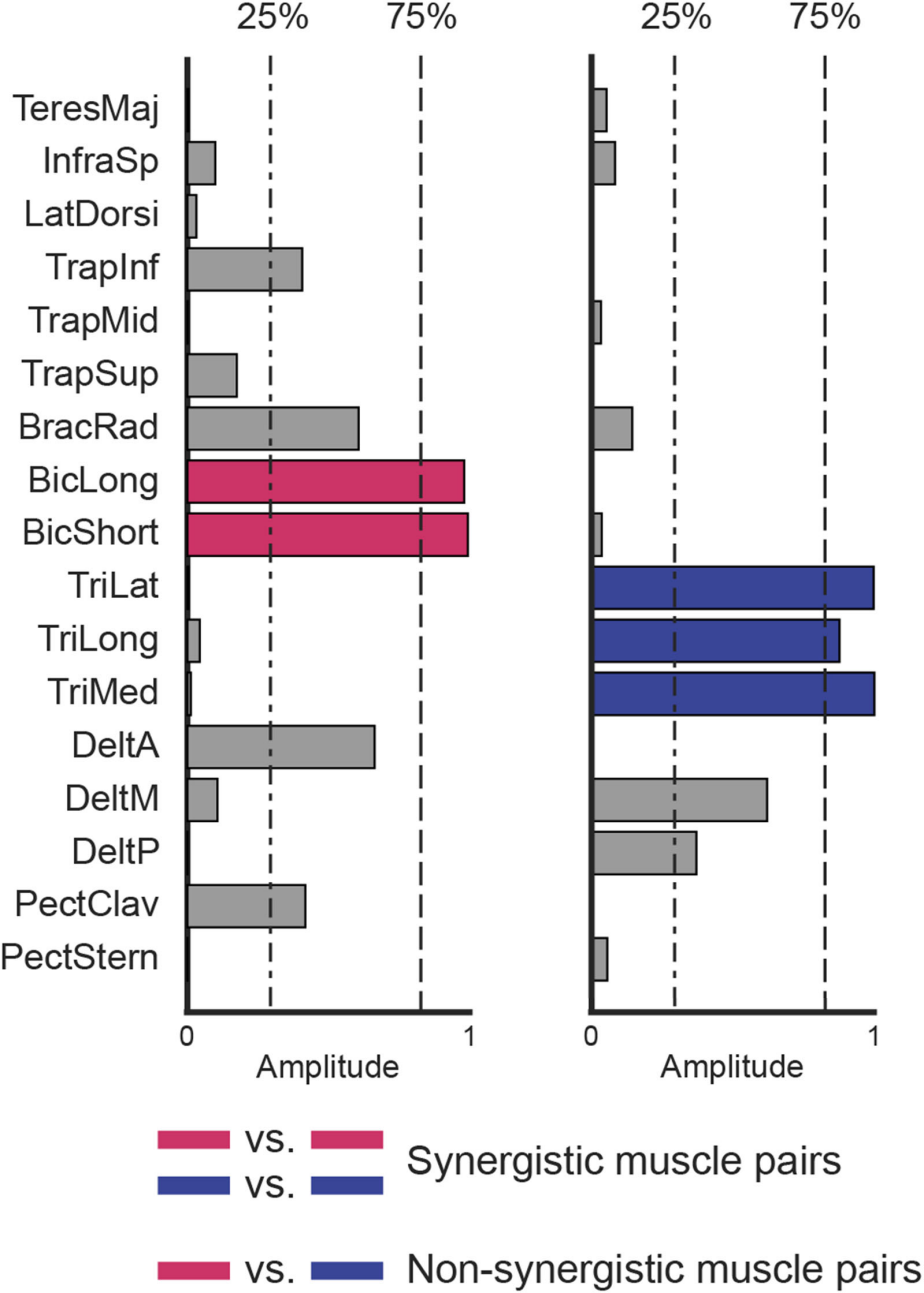
Definition of synergistic and non-synergistic muscles. Two synergies, selected in the set extracted from data collected from participant eight (synergies W3 and W6 in Figure 3), are represented. Muscles with the same color (blue vs. blue or magenta vs. magenta) are synergistic because they are exclusively recruited by the same synergy (i.e., contribution higher than 75%, dashed line). Muscles with different colors are non-synergistic (blue vs. magenta) because they are not exclusively recruited by the same synergy (i.e., if the contribution of a muscle to a synergy is higher than 75%, the contribution of the other muscle to the same synergy is lower than 25%, dash-point line).

The muscle weights (*W_m_*) were normalized across synergies, such that the squared sum of the contribution of each muscle to all synergies was set to one. Two muscles were considered to be recruited within the same synergy, and defined “synergistic muscle pair,” if they both contributed to that synergy with a normalized weight higher than 75% (Figure 2), i.e., they were both highly recruited by the same synergy. The 75% threshold was chosen to ensure that each muscle was associated with only one synergy. In contrast, two muscles were considered not to be recruited within the same synergy, and defined “non-synergistic muscle pair,” if one muscle contributed to a synergy with a normalized weight higher than 75% and the other with a normalized weight lower than 25%, or vice-versa. As the normalization approach used in this study ensured that the total contribution of each muscle across all synergies summed to 1, a muscle could exceed the 75% threshold in only one synergy. Therefore, if a pair of muscles met the “non-synergistic” criterion in one synergy, they could not be considered “synergistic” in any of the others, since the threshold condition could not be simultaneously satisfied in multiple synergies. The 25% threshold was set according to a previous study (De Marchis et al., 2013). Therefore, the intermuscular coherences calculated between all muscle pairs were assigned to the “synergistic muscle pair” category if both muscles contributed to the same synergy, or to the ‘non-synergistic muscle pair’ category if they contributed to different synergies. Pairs that did not fit either definition, e.g., cases where neither muscle showed a contribution higher than 75%, or where one muscle contributed more than 75% while the other fell between 25% and 75%, were excluded to avoid potential confounding effects.

#### 2.4.4 Subject-specific frequency layers

As the decomposition of intermuscular coherence through NMF identified different subject-specific frequency layers, i.e., the coherence patterns *W_c_*, coherence between pairs of active muscles, was analyzed within these layers. An average Z-coherence value *I_Z_* was calculated for each spectrum was calculated as in Equation 5 (De Marchis et al., 2015):


$${\text{I}}_{\text{Z}}\,\left({\text{f}}_{1},{\text{f}}_{2}\right)=\frac{1}{{\text{f}}_{2}-{\text{f}}_{1}}\,\int_{f_{1}}^{f_{2}}Z_{xy}\left(f\right)df$$ (5)

Where *f*_1_ and *f*_2_ are the lower and upper bounds of each layer. The bounds of layer *k* were identified as the frequency interval in which layer *k* showed the highest coherence with respect to the other layers, and they were subject-specific. Although *W_c_* may exhibit multiple peaks and a non-unique frequency interval, our results, consistent with previous literature, revealed a single peak, and therefore, a unique definition of the frequency interval.

We emphasize that the muscle synergy extraction and the cross-muscle coherence were not related measures. In fact, while muscle synergies described the instantaneous relative contribution in the amplitude of different muscles along different time-steps, cross-muscle coherence described the phase locking between muscles.

#### 2.4.5 Statistical analysis

We used a linear mixed model to test the effect of task and synergy on intermuscular coherence. The model included target direction (coded as separate dummy variables), repetition, and recruitment of the muscle pair by the same or different synergies as fixed factors, and participant index as a random factor. Coherence values were averaged across layers for each muscle pair. Only pairs showing significant coherence in at least one frequency bin in the (1.60) Hz interval were included, to avoid inactive muscles masking the effects. Statistical significance was assessed at *p* < 0.05.

## 3 Results

On average, 6.0 ± 4.2 trials (mean ± SD across participants) out of 160 were excluded due to EMG artifacts. All participants successfully performed the task, reaching and maintaining the required force level within a 2% MVF tolerance for 3 seconds in the vast majority of trials (number of retained trials: 152.4 ± 5.0).

### 3.1 Muscle synergies

**FIGURE 3:**
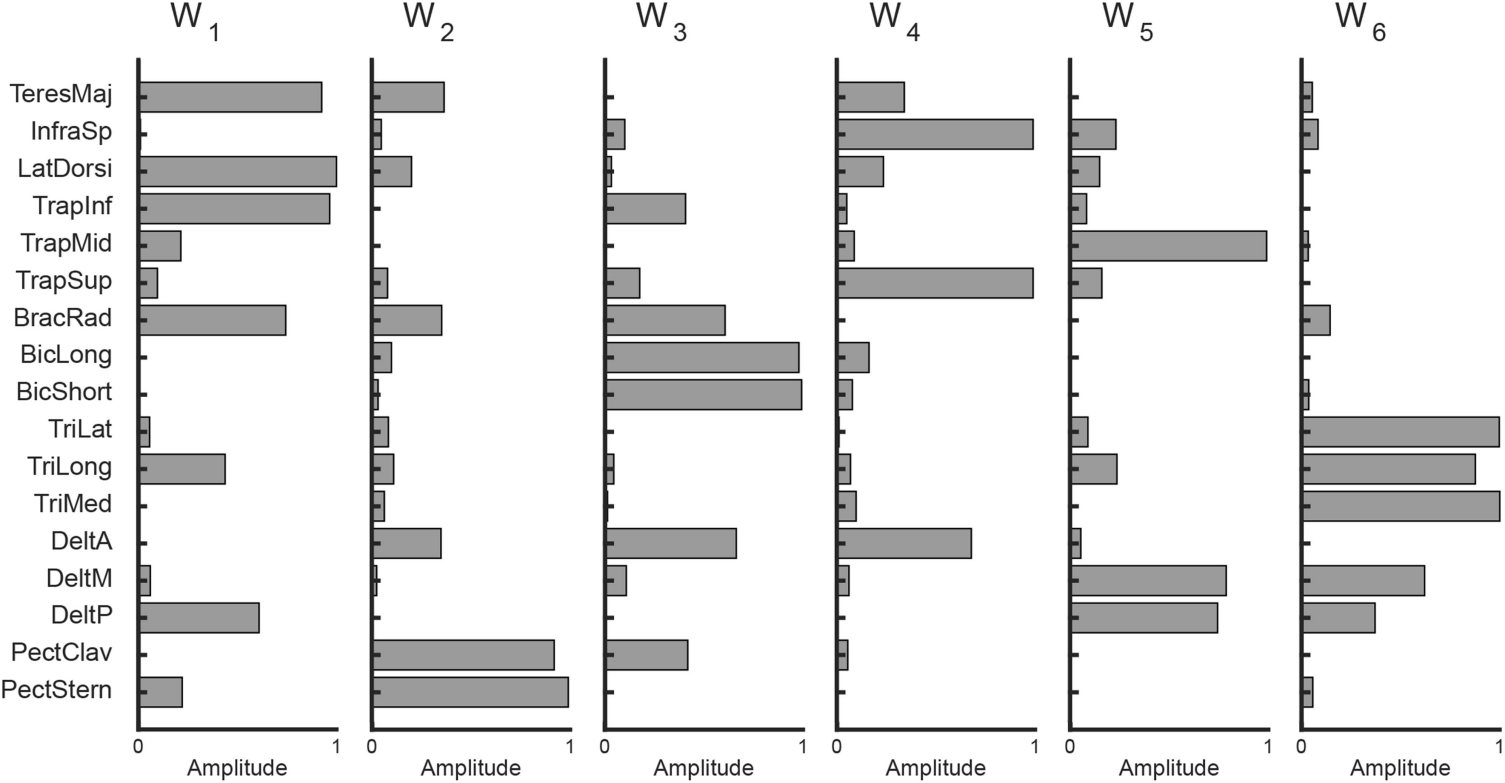
Example of muscle synergies extracted from data collected from participant eight (six synergies with *R*^2^ = 0.92).

To investigate how participants coordinated their muscles during the task, we extracted muscle synergies from the activation patterns using non-negative matrix factorization (NMF) (Lee and Seung, 1999). On average, 5.9 ± 0.6 synergies were identified across participants, which accounted for 91.4 ± 1.1% of the total data variation (*R*^2^), indicating a low-dimensional control strategy. An example of the extracted synergies from a representative participant is shown in Figure 3.

### 3.2 Intermuscular coherence: comparison within and across synergies

**FIGURE 4:**
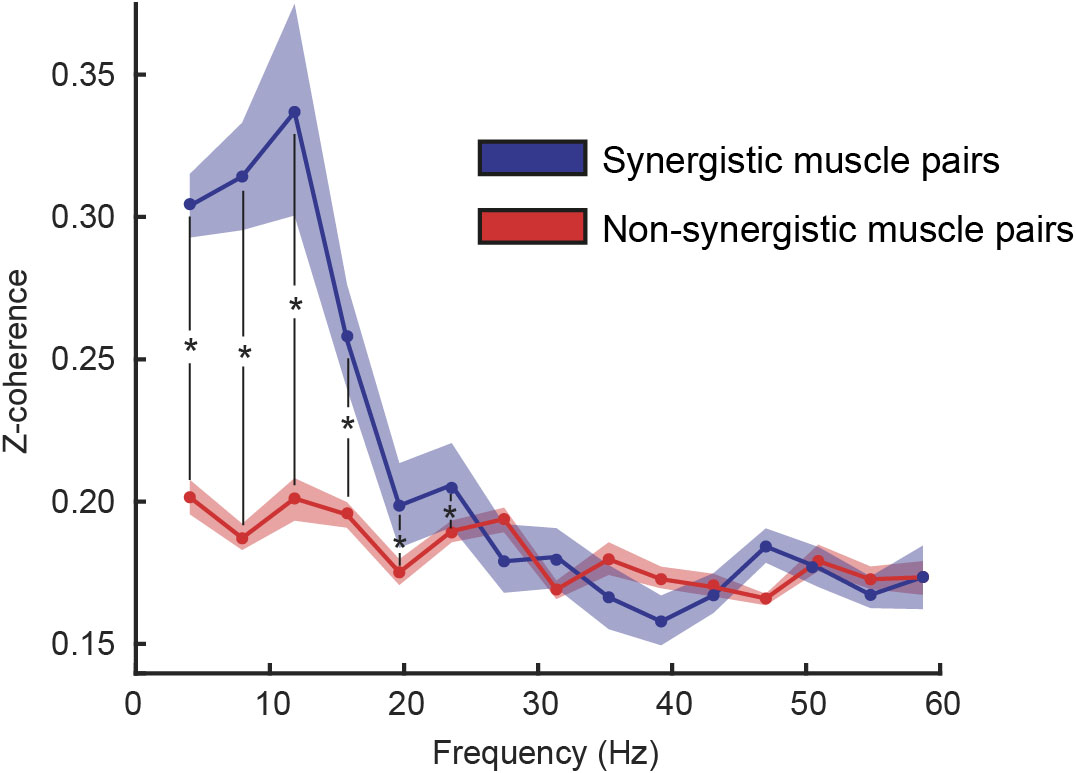
Coherence between synergistic (blue) and non-synergistic (red) muscles (mean ± standard error among participants). Results of the linear mixed model analysis identified a statistical effect (*p* < 0.05) at specific frequencies identified with a *.

To assess whether muscles recruited within the same synergy share more common neural input than muscles recruited in different synergies, we computed the intermuscular coherence across muscle pairs and compared synergistic vs. non-synergistic combinations. The linear mixed-effects model revealed significantly higher coherence for synergistic muscle pairs in frequency bins below 25 Hz (Figure 4).

However, comparing these results across studies is challenging due to differences in the definition of frequency ranges and methodological choices. To address this variability, in the following section, we applied a data-driven approach to identify subject-specific frequency layers.

### 3.3 A data-driven approach to define physiologically meaningful frequency bands

**FIGURE 5:**
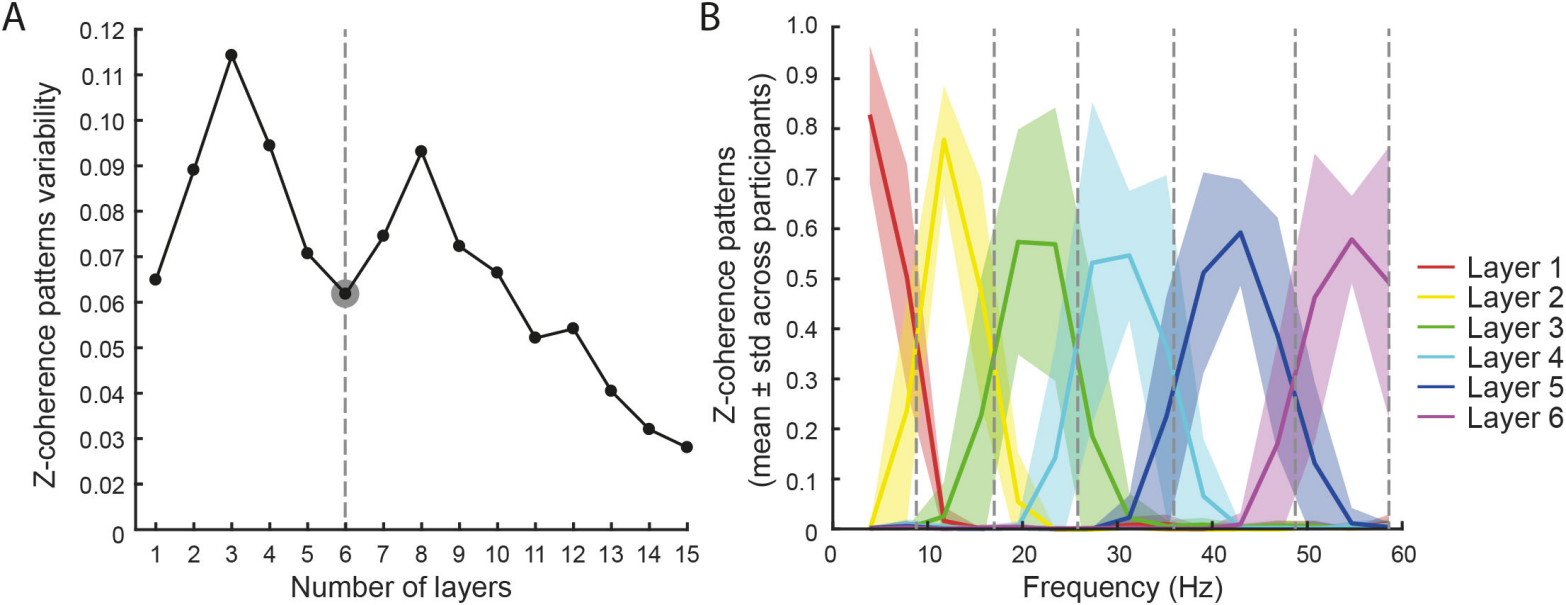
Coherence patterns. **(A)** A local minimum in the variability of the coherence pattern among participants was identified for six layers (dashed line). **(B)** The identified layers (mean ± std across participants) are plotted with different colors. The dashed lines identified the mean separation between layers.

To address this issue, we decomposed intermuscular coherence into subject-specific frequency layers. Across participants, the coherence patterns showed a consistent organization into six main layers (Figures 5A, B). Higher numbers of layers (≥11) resulted in over-discretization, splitting the spectrum into excessively narrow, physiologically meaningless bins. The subject-specific frequency boundaries identified six bands, each corresponding to a coherence layer with relatively higher intra-layer coherence (mean ± SD across participants; Figure 5B): Layer 1: [1.0 (0.0)–8.7 (1.1)] Hz; Layer 2: [8.7 (1.1)–16.9 (2.0)] Hz; Layer 3: [16.9 (2.0)–25.7 (2.9)] Hz; Layer 4: [25.7 (2.9)–35.8 (2.5)] Hz; Layer 5: [35.8 (2.5)–48.6 (2.8)] Hz; Layer 6: [48.6 (2.8)–60.0 (0.0)] Hz.

### 3.4 Coherence differences across layers for synergistic and non-synergistic muscle pairs

**FIGURE 6:**
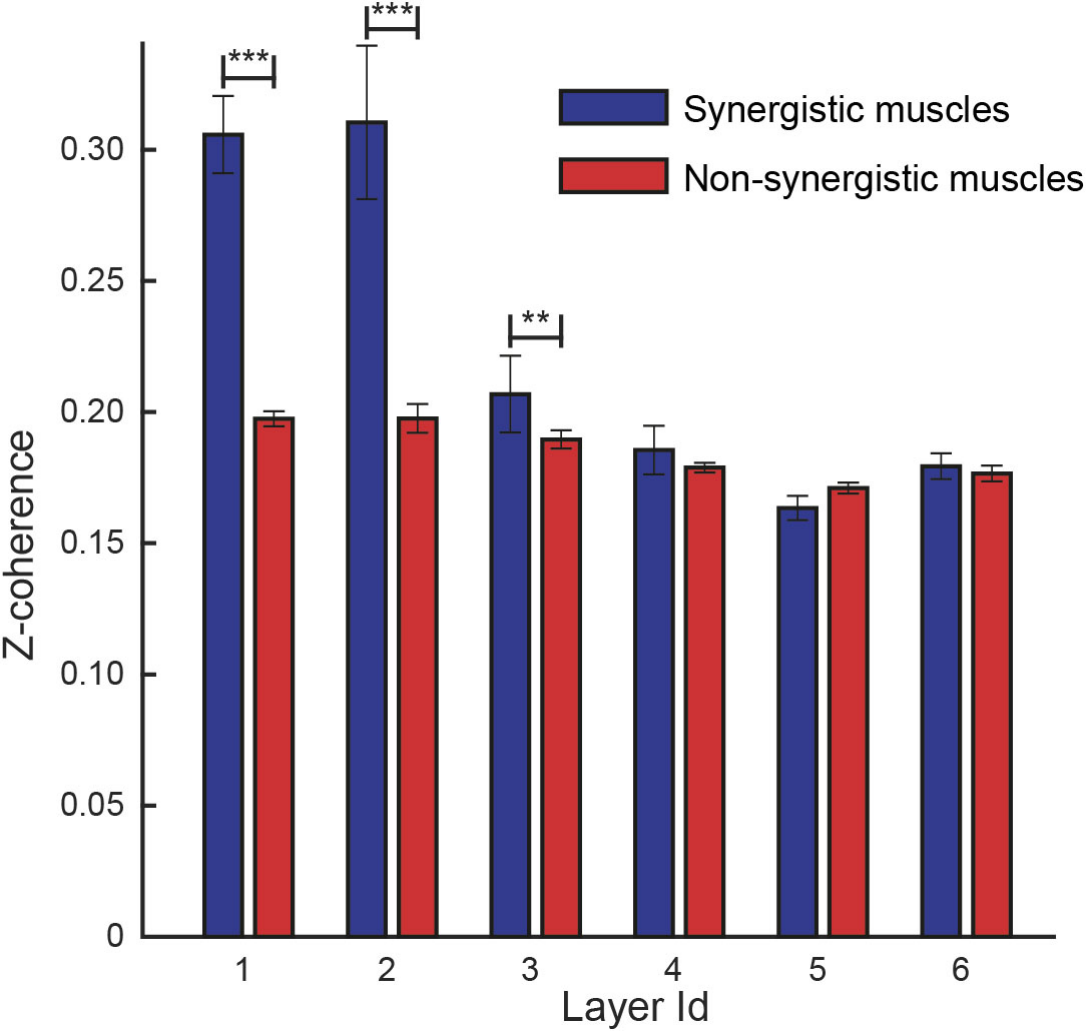
Comparison between the coherence identified on different layers between synergistic (blue) and non-synergistic (red) muscle pairs. The linear mixed model analysis identified a statistical effect of the recruitment of pairs of muscles by the same synergy in layers 1, 2, and 3. ***, *p* < 0.001; **, *p* = 0.01.

Finally, using these automatically identified layers, we re-evaluated coherence differences between synergistic and non-synergistic muscle pairs within each frequency band. The analysis included 9,486 muscle pairs (mean ± SD across participants: 1,186 ± 229, see Table 1) classified as synergistic and 45,512 as non-synergistic (5,689 ± 1,160). The linear mixed-effects model confirmed significantly higher coherence for synergistic pairs in the first three layers (*p*-values: < 0.01 for all three bands), while no significant differences were found in the higher frequency layers (*p* > 0.05 for layers 4–6; see Figure 6). These results support the hypothesis that low-frequency coherence is a marker of shared neural input within muscle synergies and demonstrate that the proposed frequency decomposition yields physiologically interpretable findings.

**TABLE 1:** Per-participant counts of synergistic and non-synergistic muscle pairs used in the linear mixed-effects analysis.

Subject id	1	2	3	4	5	6	7	8	Mean ± SD
Synergistic pairs	1,350	1,179	1,353	966	920	1.451	1.376	891	1.186 ± 229
Non-synergistic pairs	5.171	4.426	6.296	4.764	4.286	7.151	6.985	6.433	5.689 ± 1.160

## 4 Discussion

We demonstrated that, during a submaximal multidirectional isometric force generation task, pairs of muscles recruited within the same synergy show a higher degree of coherence with respect to pairs of muscles recruited by different synergies, in the delta-theta (1st layer), alpha (2nd layer), and low-beta (3rd layer) frequency bands.

### 4.1 A novel approach to determine functionally relevant frequency layers

The automatic separation, obtained with the decomposition of intermuscular coherence, identified spectral layers that are consistent with functional frequency bands reported in the literature.

Different EMG frequency components are known to reflect distinct sources of common drives to the motor neurons and to different functional roles in motor control (MacKay, 1997; Brittain and Brown, 2014; Ramos-Murguialday and Birbaumer, 2015; Leonardi et al., 2022), and our findings are consistent with this framework. The low-frequency components of the neural drive, i.e., delta and alpha bands, are likely of spinal origin (Farina and Negro, 2015) and represent the effective drive to muscles for force generation (Negro et al., 2009; Farina et al., 2014). Contrarily, higher frequency components are effectively filtered by the muscle dynamic (Negro et al., 2009), but are found in cortico-muscular coherence, thus suggesting to derive from the rhythmic discharges of the corticospinal neurons projecting to the spinal motoneurons (Farmer et al., 1997; Mima and Hallett, 1999). Significant cortico-muscular coherence has been identified in the beta band during isometric force production tasks (Kristeva-Feige et al., 2002), and in higher frequency bands, i.e., the gamma band, during dynamic conditions (Omlor et al., 2007). Importantly, the present results support and extend this view by identifying frequency layers that partially overlap with these functional subdivisions. In the last decade, a further functional subdivision of the beta band into a low- and high-frequency range hinted at a contribution of the cortico-basal ganglia indirect pathway to low-beta oscillations and the hyperdirect pathway for high-beta oscillations (Oswal et al., 2016; Milardi et al., 2019), leading to the separate investigation of these bands (van Wijk et al., 2017; Plate et al., 2021). Notably, our decomposition revealed finer substructure within the beta range, consistent with this functional split, thus suggesting that the identified coherence layers may reflect different sources of cortical modulation. Similarly, the gamma band was separated into its low- and high-frequency components (Hermer-Vazquez et al., 2007; Uhlhaas et al., 2011), and our approach may further help distinguish these subcomponents in a subject-specific manner. This data-driven identification of frequency layers therefore bridges the gap between conventional, literature-based frequency bands and subject-specific spectral organization. Thus, this approach would improve comparability across subjects and may prove advantageous in patient studies where spectral content is altered (Houston et al., 2021).

Unlike previous studies that relied on predefined frequency boundaries, e.g., alpha up to 12–15 Hz, low-/high-beta split around 20–25 Hz, or beta–gamma transitions up to 35 Hz (Mehrkanoon et al., 2014; De Marchis et al., 2015; Del Vecchio et al., 2019; Leonardi et al., 2022), our approach derives such divisions directly from participant data. Intriguingly, the frequency layers identified by decomposing the intermuscular coherence with NMF were in line with the physiologically meaningful frequency bands reported in previous literature, such as those representing the effective drive for force generation (Negro et al., 2009) or those hypothesized to have a cortical origin as identified in the cortico-muscular coherence (Mima and Hallett, 1999). In particular, the 1st layer (mean (std) ([1.0 (0.0) 8.7 (1.1)] Hz) resampled the delta and theta bands, the 2nd layer ([8.7 (1.1) 16.9 (2.0)] Hz) the alpha band, the 3rd layer ([16.9 (2.0) 25.7 (2.9) Hz]) the low-beta band, the 4th layer ([25.7 (2.9) 35.8 (2.5)] Hz) the high-beta band, the 5th layer ([35.8 (2.5) 48.6 (2.8)] Hz) the low-gamma band, and the 6th layer [48.6 (2.8) 60.0 (0.0)] Hz) the high-gamma band. The use of subject-specific bands detected from participant data, on one hand, overcomes the lack of a unique separation of relevant bands, and on the other hand, enables the study of neurological patients with altered muscle activation patterns, such as stroke survivors (Houston et al., 2021).

### 4.2 Muscles recruited within the same synergy show higher coherence in the delta, alpha, and low-beta frequency bands

The present findings extend previous attempts to link the muscle synergy framework with the concept of common synaptic input to motoneurons (Danna-Dos-Santos et al., 2014; De Marchis et al., 2015; Frère, 2017; Ortega-Auriol et al., 2019; Laine et al., 2021; Borzelli et al., 2024b). By characterizing coherence patterns across a wide range of frequencies and a large set of muscles, we demonstrated that synergistic structures are reflected in frequency-specific common drives, bridging functional and neural descriptions of motor coordination. This supports the view that muscle synergies may emerge from shared neural inputs distributed across multiple motoneuron pools.

In line with previous evidence, we demonstrated that muscles recruited within the same synergy (synergistic muscle pairs) showed a higher coherence with respect to muscles recruited by different synergies (non-synergistic muscle pairs) in the low-frequency layers, likely involved in force generation (i.e., the 1st and 2nd layers representing the delta-theta and alpha bands) (Ortega-Auriol et al., 2019; Laine et al., 2021). However, we also identified a higher coherence between synergistic muscles compared to non-synergistic muscles even in the 3rd layer, representing the low-beta band (Laine et al., 2015; Borzelli et al., 2024b), which is likely to be of cortical origin.

This frequency-specific pattern supports the coexistence of spinal and cortical contributions to muscle coordination: lower frequencies reflecting shared spinal drives for force generation, and low-beta coherence suggesting a supraspinal descending component.

Compared to previous studies (Ortega-Auriol et al., 2019; Laine et al., 2021), our analysis extends these findings to a larger set of muscles and a broader frequency range, revealing a more continuous organization across layers rather than discrete bands. The subject-specific decomposition thus refines the identification of relevant frequencies without imposing arbitrary boundaries, enhancing sensitivity to subtle spectral peaks and inter-individual variability.

Together, the coexistence of low-frequency and low-beta coherence refines the current view of spinal and cortical drives. Specifically, the low-frequency layer likely reflects shared spinal inputs coordinating force generation, while the low-beta layer indicates a cortical contribution that modulates fine control and adaptation. This coexistence suggests that spinal and cortical mechanisms may jointly shaping muscle coordination within synergies.

Moreover, the subject-specific frequency-layer approach provides an advance over conventional fixed-band analyses by aligning the identified coherence peaks across individuals. This data-driven segmentation reduces the bias introduced by arbitrary frequency boundaries and allows the detection of subtle, participant-specific spectral features that would otherwise be masked in averaged, pre-defined frequency bins.

Significant coherence between the activity of different muscles represents a signature of the common input that drives these muscles. While the identified significant low-frequency coherence suggests that the spinal drive, which regulates the muscle activation, is shared across different muscles, the occurrence of a significant coherence also in the low-beta band suggests the existence of a supraspinal common descending cortico-spinal drive, in line with studies on cortico-synergistic coherence (Zandvoort et al., 2019, 2022; Ortega-Auriol et al., 2023). As multiple joints and muscles are coordinated by spinal premotor circuits (Takei and Seki, 2010; Takei et al., 2017), we may hypothesize the existence of separate cortical inputs, modulating a network of spinal premotor interneurons (Song et al., 2022) that, in turn, modulates the firings of subpopulations of the MNs of muscles composing a synergy (Hug et al., 2022). Further investigations will validate this hypothesis.

### 4.3 Applications

These findings have potential translational implications for clinical and neuroengineering applications.

Because neurological patients often exhibit altered synergy organization (Roh et al., 2013; Mileti et al., 2020; Houston et al., 2021), the identification of distinct low-frequency and low-beta layers provides potential biomarkers of spinal and cortical contributions to coordination. Quantifying changes in these frequency-specific coherence patterns may therefore offer a sensitive marker of disease severity and motor recovery.

Beyond clinical diagnostics, specific frequency bands may also be leveraged to enhance the control of myoelectric devices. EMG activity has already been explored as a control signal for robotic devices (Ajoudani et al., 2012; Song et al., 2023), exoskeletons (Durandau et al., 2019; Borzelli et al., 2020; Caggiano et al., 2022), prostheses (Cimolato et al., 2022; Yadav and Veer, 2023), and motor augmentation systems (Gurgone et al., 2022; Lee et al., 2024; Lisini Baldi et al., 2025). Notably, the beta-band firing of individual motor units has been successfully implemented in controlling virtual cursors for human augmentation (Bräcklein et al., 2021), consistent with our identification of a low-beta layer reflecting cortical drive, by targeting the frequency components most relevant for either spinal (force) or cortical (fine control) drives. Thus, the present frequency-layer framework may inform future closed-loop strategies that adaptively exploit specific coherence bands for neurorehabilitation and assistive technologies.

### 4.4 Limitations

In this study, the number of layers was determined according to the consistency among participants. While this approach is reasonable for healthy individuals, it may not be suitable for neurological patients, who often exhibit significant pathological variability (Houston et al., 2021).

Similar issues may arise during complex tasks, e.g., when the modulation of limb stiffness is required together with a force generation task (Borzelli et al., 2018, 2023; Gurgone et al., 2022). While simple one-degree-of-freedom models based on two antagonist muscles have successfully described the relationship between coactivation and joint stiffness (Borzelli et al., 2017a, b), such formulations cannot capture the multidimensional nature of muscle coordination and stiffness modulation observed in multi-muscle, multi-directional tasks. Therefore, different muscle patterns may be exploited to achieve the same limb stiffening level, and consistency among participants may not be detected. Further studies are needed to investigate the changes in synchronous muscle modulation during combined tasks involving force generation and limb stiffness modulation.

The coherence analysis requires a static signal, so dynamic tasks would require alternative time-frequency analyses (Di Nardo et al., 2022; Borzelli et al., 2025). However, the availability of a dynamic task, e.g., an isotonic task, together with an isometric task, like the one presented in this study, would provide a comprehensive description of the spectral features of muscles recruited by the same synergy.

A further limitation concerns the disparity in the number of synergistic and non-synergistic pairs. Although the two groups were unbalanced, the linear mixed-effects model included participant index as a random factor, thus controlling for inter-individual variability and preventing group size differences from biasing the estimated effects.

Finally, while our subject-specific frequency-layer approach improves inter-subject comparability and highlights physiologically meaningful coherence bands, future work should test its robustness in patient populations with highly variable spectral content.

## 5 Conclusion

We demonstrated that a subject-specific, data-driven decomposition of intermuscular coherence reveals physiologically relevant frequency bands underlying muscle synergies.

Compared with classical fixed-band analyses, this approach refines the detection of frequency-specific coherence and offers a framework to study altered motor coordination in patient groups.

These findings support the hypothesis that the coordination of different muscles in muscle synergies is implemented at the neural level through shared neural drives at both spinal and cortical frequencies.

## Data Availability

The raw data supporting the conclusions of this article will be made available by the authors, without undue reservation.
